# Reporting back environmental exposure data and free choice learning

**DOI:** 10.1186/s12940-015-0080-1

**Published:** 2016-01-09

**Authors:** Monica D. Ramirez-Andreotta, Julia Green Brody, Nathan Lothrop, Miranda Loh, Paloma I. Beamer, Phil Brown

**Affiliations:** Department of Soil, Water, and Environmental Science, University of Arizona, 1177 E Fourth Street, Rm. 429, Tucson, Arizona USA; Silent Spring Institute, Newton, MA USA; Mel and Enid Zuckerman College of Public Health, University of Arizona, Tucson, AZ 85721 USA; Institute of Occupational Medicine, Edinburgh, UK; Department of Sociology and Anthropology and Department of Health Sciences, Northeastern University, Boston, MA USA

**Keywords:** Biomonitoring, Exposure assessment, Environmental health literacy, Risk communication, Arsenic, Heavy metals, Informal science education, Free-choice learning

## Abstract

Reporting data back to study participants is increasingly being integrated into exposure and biomonitoring studies. Informal science learning opportunities are valuable in environmental health literacy efforts and report back efforts are filling an important gap in these efforts. Using the University of Arizona’s Metals Exposure Study in Homes, this commentary reflects on how community-engaged exposure assessment studies, partnered with data report back efforts are providing a new informal education setting and stimulating free-choice learning. Participants are capitalizing on participating in research and leveraging their research experience to meet personal and community environmental health literacy goals. Observations from report back activities conducted in a mining community support the idea that reporting back biomonitoring data reinforces free-choice learning and this activity can lead to improvements in environmental health literacy. By linking the field of informal science education to the environmental health literacy concepts, this commentary demonstrates how reporting data back to participants is tapping into what an individual is intrinsically motivated to learn and how these efforts are successfully responding to community-identified education and research needs.

## Background

Biomonitoring efforts are widely used in environmental exposures assessments beyond occupational and clinical settings to help identify and assess chemicals observed in the environment and in humans and to inform public-health decisions and regulations [1]. Due to innovations in technology, increased sensitivity in methods, access to resources, and institutional support for community-engaged research, there has been a tremendous increase in the number of human biomonitoring studies [1, 2]. With these types of studies comes an additional level of responsibility to translate the data and findings and address environmental health literacy (EHL) goals.

Using the University of Arizona’s Metals Exposure Study in Homes (MESH) extensive report back effort as an example, this commentary highlights how reporting back environmental exposure data is carving out a new informal education setting (learning outside of school classrooms) and is stimulating free-choice learning - learning that is occurring within these settings and that is driven by the needs and interests of the learner rather than an external authority [3]. By taking an in-depth look at exposure study participants’ understanding of results and their resulting actions, this commentary contributes to exposure science and expands the concept of EHL and science education by viewing report back as a free-choice learning experience. Lastly, it is proposed that such documentation of learning and action would be an effective method by which to assess report back efforts and, in effect, evaluate a community’s EHL.

### Literacy

Thus far, scholars have defined literacy in terms of science [4], health [5], critical health [6], public health [7], and the environment [8]. Health literacy implies the achievement of a level of knowledge, personal skills and confidence to take action to improve personal and community health by changing personal lifestyles and living conditions [5]. Public health literacy is defined as the degree to which individuals and groups can obtain, process, understand, evaluate, and act on information needed to make public health decisions that benefit the community [9]. Environmental literacy is the capacity for individuals and groups to make informed decisions concerning the environment; to be willing to act on these decisions to improve the well-being of other individuals, societies, and the global environment; and to participate in civic life [8]. Recently, efforts have sought to merge existing explanations and define EHL. Thus far, the Society for Public Health Education defines EHL as the ability to “integrate concepts from both environmental literacy and health literacy to develop the wide range of skills and competencies in order to seek out, comprehend, evaluate, and use environmental health information to make informed choices, reduce health risks, improve quality of life and protect the environment” [10].

Literacy as described above, stresses understanding, informed decision-making, and action. However, health literacy efforts traditionally assess basic literacy skills such as reading, writing, and arithmetic, and are geared toward compliance with recommended clinical care. In this context, health literacy is seen as a patient “risk factor” that needs to be managed within the process of providing clinical care [11] and tends to stay within the traditional disciplinary boundaries of medicine and health promotion [6]. This approach does not view health literacy as an asset or a form of health action (personal, social, environmental) and does not focus on or assess individual and/or community efforts regarding knowledge integration, informed choices, actions to improve personal and community health, and methods to reduce risk. When health literacy is viewed as an asset, with roots in public health [11], practitioners can then implement and evaluate “health literacy in action.” This is not only defined by functional literacy, but also communicative/interactive and critical literacy, which can then assess whether an individual/community is taking action to improve health, exerting greater control over factors that determine health, reflecting greater autonomy and empowerment, and engaging in a wider range of health actions [6, 11]. These actions include personal behaviors to social action to address the social, economic, and environmental determinants of health. EHL is an evolving concept that spans and synergistically unites various disciplines such as, but not limited to, risk communication, social science, public health, health promotion, and communication [12]. Interestingly the field of science education, specifically the subfield of informal science learning/free-choice learning, has not been part of the dialogue thus far, and this is a great loss. Efforts to understand how, when and where learning is occurring are at the forefront of informal science education efforts. Measuring these types of changes can be challenging and new methods are needed to improve and evaluate literacy in action, specifically EHL.

Informal science education, defined as science learning opportunities that people experience across their lifespan outside of school [13], contributes greatly to an adult’s science learning. In fact, over the course of a lifetime, the average person spends 5 % of their time in school [14] and research suggests that nearly half of the public’s science understanding and learning derives from free-choice learning [13]. In a study to determine what sources people relied upon for science and technological information, 74 % of respondents attributed “some or a lot” of their learning to “life experiences,” followed by “books and magazines –not for school” [15]. Data indicates that lifelong learning is intrinsically motivated and largely under the choice and control of the learner [13, 15, 16]. It seems ideal to harness the power of free-choice learning to improve EHL.

### Free-choice learning in environmentally compromised communities

Informal science education methods are particularly valuable when working with communities impacted by hazardous waste. Communities neighboring contaminated sites are learning on their own about the contaminants of concern at hazardous waste sites and the associated health effects, and this type of learning is a multi-faceted phenomenon that is place-based and socioculturally mediated. As suggested by Falk and Storksdieck (2005), free-choice learning is a cumulative process involving connections and reinforcement among the variety of learning experiences people encounter in their lives [17]. In that way, learning is both a process and a product, suggesting a vibrant and contextual model of learning [17], requiring both creative and innovative evaluation methods [18].

## Community-engaged exposure assessment studies as informal education settings

There is already an established infrastructure of organizations providing opportunities for free-choice learning beyond and outside of the formal education system. These include broadcast and print media, libraries, museums, science centers, zoos, aquariums, botanical gardens, environmental centers, and community organizations [13]. Although environmental health issues are discussed broadly and may be addressed in the above organizations, there are no infrastructures dedicated to environmental health, particularly for discussing contaminant fate and transport, exposure assessments, risk characterization, and the challenges of establishing a relationship between exposure(s) and health outcome(s). These topic areas make EHL and free-choice learning especially important to communities neighboring hazardous waste, pollution emissions, and resource extraction activities. Residents of environmentally compromised communities typically want additional information regarding their environmental health, and due to this gap in infrastructure, such communities have begun partnering with research organizations and universities to conduct community-engaged research projects [19, 20]. These partnerships are establishing new informal education infrastructures that support and stimulate free-choice learning. Collaborative research efforts are increasingly directed toward understanding the environmental determinants of chronic disease, and biomonitoring is becoming a key methodology by which to provide the scientific basis for prevention of environmental exposures and motivating action [21].

### The need for report back efforts in mining communities

Evaluating exposures and increasing EHL in mining communities is especially crucial. Mining and smelting activities are the primary source of metals entering the environment [22]. In the U.S. alone, approximately 550,000 abandoned mine sites are responsible for generating 45 billion tons of waste, and many of these sites are in arid and semiarid regions [23]. Studies have observed an inverse relationship between the environmental and biomonitoring levels of arsenic and heavy metals and the distance from metal smelters and other mining operations to home or school environments [24, 25]. In an exposure study conducted near the Tar Creek Superfund Site, a former lead and zinc mine, half of the homes sampled had indoor dust concentrations of arsenic, lead, cadmium, and zinc greater than those observed in soil [26]. Due to their common occurrence at hazardous waste sites, toxicity, and potential for human exposure, metals like arsenic, cadmium, and lead are among the Agency for Toxic Substances and Disease Registry’s top ten contaminants of concern [27]. Particulate emissions associated with mining operations are commonly associated with significantly elevated levels of one or more of these contaminants [25, 28].

In Arizona alone, there are more than 80,000 abandoned mines [23] and the arid and semi-arid Southwest climate creates great potential for dust emissions and long-range transport of arsenic-contaminated aerosols from these former mining operations [29]. Climate change will exacerbate the risks posed by mining in arid and semi-arid environments, primarily due to land use changes, increased average temperatures, and drought conditions [30]. The Town of Dewey-Humboldt, Arizona, comprised of 3,894 people [31], is sandwiched in between a 153-acre site of legacy mine tailings waste with arsenic and lead concentrations exceeding 3,000 milligram per kilogram and a 189-acre legacy smelter area [32]. Dewey-Humboldt, AZ is home to the Iron King Mine and Humboldt Smelter Superfund Site, a hazardous waste site added to the US Environmental Protection Agency’s National Priorities List in 2008. Soon after listing, members of the Dewey-Humboldt, AZ community partnered with the University of Arizona (UAZ) on community-engaged environmental health research initiatives to characterize the extent of anthropogenic and naturally occurring of arsenic and metal contamination in their residential areas (e.g., Gardenroots, [33–35]). The Metals Exposure Study in Homes (MESH) project was developed in response to community concerns about exposure to metal(loid)s such as arsenic, lead, cadmium, nickel, beryllium, aluminum, and chromium. MESH assessed metal concentrations in multiple environmental matrices and their associations with levels of exposure in local children (1–11 years of age) [36]. After the completion of the MESH study and data report back efforts, participants were asked to reflect upon the report back experience, their understanding of results, and actions in response to their results. Not surprisingly, MESH participants described activities that are aligned with the EHL goals. Participants used the MESH data to inform what actions to take to reduce their child’s exposure; they posed new research questions; and suggested novel ideas on how to report the data to inform them even further. These outcomes reflect participants’ ability to comprehend, evaluate, and use the provided environmental health information to make informed choices and reduce health risks – meeting the EHL literacy goals defined above. Challenges described by the MESH participants included: access to and networking with other participants, more face-to-face engagement with the research team, additional information related to all the metals analyzed, and a spatial representation of the data. Though MESH participants described challenges associated with the report back process, the outcomes of the project are still aligned with EHL goals. Practitioners and researchers can learn from the reported challenges to improve their future report back efforts. These outcomes reflect the findings of past studies designed to take in-depth look at participants’ report back experience, their understanding of results, and actions in response to their results [21, 35, 37–40]. These past studies have shown that participants can: learn a great deal [37]; interpret scientific results to affirm lay knowledge; absorb new information regarding other pollutant sources; and gain an understanding of the complex health messages related to environmental quality [35, 38]. Participants can also increase their understanding of environmental science and the scientific method; establish new networks; participate in other environmental projects; and leverage their results to hold government officials to more stringent cleanup standards [35].

## Evaluating the outcomes of reporting back environmental exposure data

By evaluating the outcomes of report back efforts, new ways to assess EHL can be illuminated, providing a wider set of parameters to more adequately and successfully reflect health literacy. MESH was one of eight biomonitoring studies selected as part of the Personal Exposure Report-Back Ethics Study, a larger project that examines how: a) researchers report back data; b) Institutional Review Boards evaluate such protocols; and c) participants understand and use results. The PERE research team is among the first to report levels of emerging (i.e. limited toxicological data) and known contaminants to individuals and communities and is leading the field of report back practices and methodologies [21, 35, 37–40]. Frameworks such as clinical ethics, community based participatory approach, and the exposure experience have been applied in the past for analyzing participant responses [21, 38, 39, 40]; based on this work, a set of best practices have been developed to guide academic-community research collaborations [39]. The exposure experience, which builds on the concept of “illness experience” [38], considers how: 1) individuals, communities, and populations are becoming increasingly aware of environmental issues, especially those related to their immediate environment, and are learning about contaminants in their bodies; and 2) the eco-social context, which encompasses participants' past experiences with pollution and how those frame their responses and actions [37]. We know that the distribution of environmental pollution varies across populations and places [41], and these geographic and social differences, and even differences in contaminants of concern, will inform their responses to data about chemicals in their homes and bodies.

In the case of Dewey-Humboldt, AZ, some community members take pride in the mine, which is a large part of their eco-social context. Even the town seal has a graphical depiction of the mine and metal smelter, alongside an agricultural field and the town hall. These idiosyncrasies need to be considered when designing report back efforts and evaluating their effectiveness to raise EHL and see positive changes at individual, programmatic, and community/social-ecological level. For example, a community member who has lived in Dewey-Humboldt her whole life states: “Well I would [have concerns] if we had like a chemical plant, or something like that around here. But, I mean, just the mine. That mine’s been there forever.” In a recent assessment at the Iron King Mine and Humboldt Smelter Superfund Site after MESH and “Gardenroots: The Dewey-Humboldt Arizona Garden Project” [33–35], Ramirez-Andreotta et al. (2015): evaluated community inquiries at the time the site was listed and five years later; assessed what community members were most concerned about at a the site; whether these concerns changed; and if these concerns were adequately addressed through accessible sources of information. Further, it was hypothesized that the changes in concern and inquiry over time would demonstrate a progression of the community’s environmental health understanding (transitioning from knowledge acquisition to application) as a result of the community involvement and engaged research efforts [42]. Key findings of this study were that a cross-sectional analysis at multiple points in time can better describe the social–ecological developments within a community and that changes in concern can demonstrate broader community recognition of the fundamentals of environmental health research (i.e., from understanding the source to potential exposure pathways and exposure mitigation) [42]. Researchers showed how community concerns changed over time as a result of the US Environmental Protection Agency's outreach and UAZ’s community-engaged research activities; and that such documentation at contaminated sites is a novel method in which to assess EHL efforts.

## Conclusions

A recent national study observed that 29 % of former lead smelters are located in areas prone to natural disasters (floods, earthquakes, tornadoes, and hurricanes), and these locations are at high risk for (re)dispersing toxic chemicals during such an event [43]. The recent Gold King Mine Spill near Silverton, Colorado, where three million gallons of water and waste (e.g. cadmium, copper, lead, arsenic) were inadvertently released into Cement Creek, a tributary of the 126-mile long Animas River [44], reminds us that these mining sites and the wastes they contain are not latent. Legacy mining sites may appear to be dormant, and it is crucial to work with communities to characterize the fate and transport of pollutants off site and to report exposure assessment data back to residents in order for them to translate the results into action. Using the MESH study as an example, this commentary contributes to exposure science and expands the concept of EHL and science education by describing how community-engaged partnerships are creating a new informal learning setting, where report back efforts are supporting free-choice learning. In these newly carved out settings, free-choice learning is occurring at the local level and is a product of conversations taking place between environmental health researchers and community members regarding their personal exposure data. These efforts are building a foundation for a sustainable and informal learning continuum, while meeting the most crucial steps in EHL efforts – empowerment, intervention, and increasing awareness. Free-choice learning is responsible for over 50 % of an adult’s learning - it is time for EHL efforts to recognize the role of free-choice learning and to build upon the intrinsic motivation that individuals have to ultimately protect their environmental health.

## Abbreviations

EHL: Environmental Health Literacy
MESH: Metals Exposure Study in Homes
UAZ: University of Arizona
